# Peliosis hepatis disseminated rapidly throughout the liver in a patient with prostate cancer: a case report

**DOI:** 10.1186/s13256-015-0682-9

**Published:** 2015-09-12

**Authors:** Hisashi Hidaka, Makoto Ohbu, Takahide Nakazawa, Takaaki Matsumoto, Akitaka Shibuya, Wasaburo Koizumi

**Affiliations:** 1 Department of Gastroenterology, Internal Medicine, Kitasato University School of Medicine, 1-15-1 Kitasato, Minami-ku, Sagamihara, Kanagawa 252-0374 Japan; 2 Department of Pathology, Kitasato University School of Allied Health Science, Sagamihara, Japan

## Abstract

**Introduction:**

In the World Health Organization histological classification of the liver tumor, peliosis hepatis is defined as a tumor-like lesion. The entity is characterized by the appearance of multiple cyst-like, blood-filled spaces within the liver parenchyma.

**Case presentation:**

A 77-year-old Japanese man with prostate cancer was referred to our department because he was diagnosed as having two hepatic tumors. The tumors were confirmed to be peliosis hepatis by repeated needle biopsies and because of their atypical images by enhanced computed tomography and enhanced magnetic resonance imaging. Later these tumors grew rapidly, increased in number, and disseminated throughout his whole liver. We are now treating the patient conservatively due to his age and his existing medical conditions.

**Conclusion:**

Peliosis hepatis is a rare hepatic benign tumor that should be considered in the differential diagnosis of multiple unknown liver tumors that are revealed by atypical radiological images.

## Introduction

When a patient presents with multiple liver tumors revealed by atypical radiological images, a decision as to whether liver needle biopsies are necessary must be carefully made because needle biopsies can cause serious complications [1]. In the World Health Organization (WHO) histological classification of the liver tumor, peliosis hepatis (PH) is defined as a tumor-like lesion. The pathological entity is characterized by the appearance of multiple cyst-like and blood-filled spaces within the liver parenchyma [2, 3]. It is currently considered that PH is related to human immunodeficiency virus (HIV) infection [4], agents administered after kidney transplantation [5], and the administration of oral contraceptives [6]. Here we report a case of PH that rapidly disseminated throughout the whole liver in a patient with prostate cancer.

## Case presentation

A 77-year-old Japanese man was referred to our department because he was diagnosed as having hepatic tumors. His history included an old myocardial infarction, obstructed arterial sclerosis of his bilateral lower feet, and prostate cancer. In February 2010, he had a luteinizing hormone-releasing hormone (LH-RH) agonist injection and radiation therapy for prostate cancer for 1 year. In March 2012, he was found to have liver tumors for the first time during a regular follow-up abdominal ultrasonography.

**Fig. 1:**
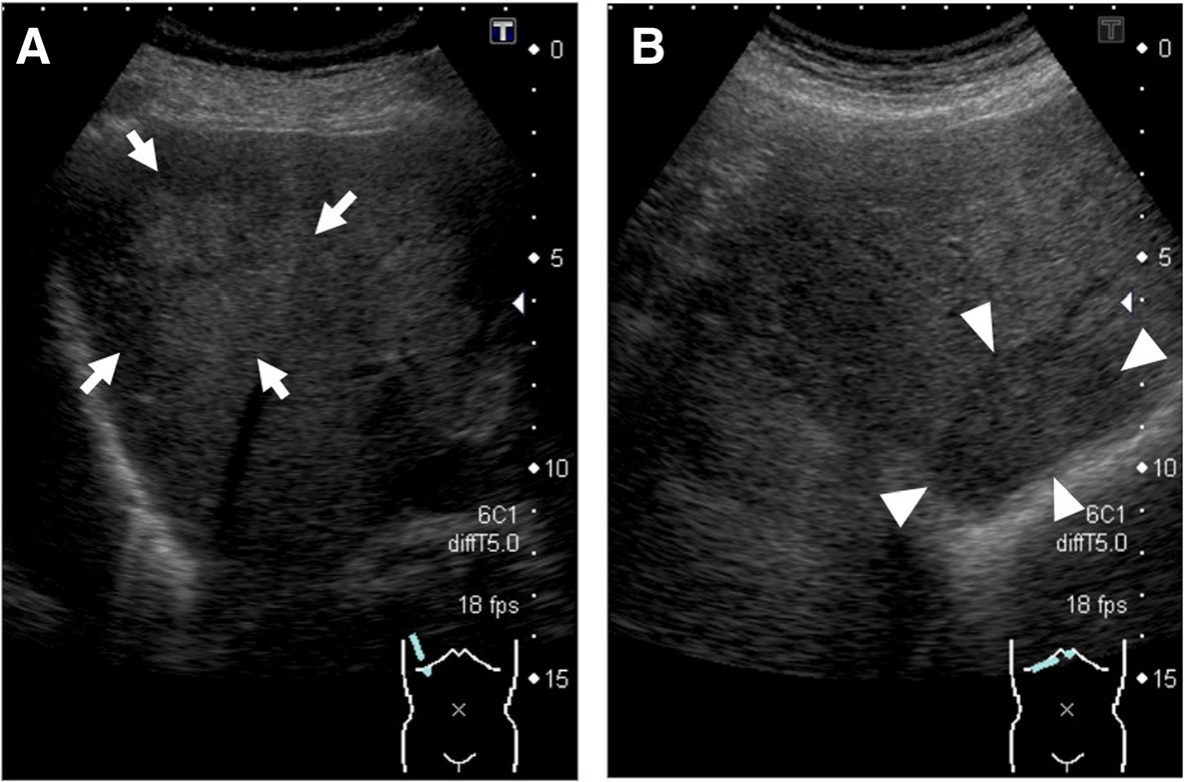
Abdominal ultrasonography shows two hepatic tumors in March 2012. **a** A high echoic tumor (50×36mm) with an unclear margin was observed in segment 7 (*white arrows*). **b** A low echoic tumor (30×18mm) with a clear margin was observed in segment 6 (*white arrowheads*)

He had no history of tobacco smoking or drinking alcohol and no family history of liver disease, hypertension, diabetes mellitus, or liver malignancy. He had been asymptomatic; however, two liver tumors were shown in an ultrasonographic examination image: 50×36mm in segment (S) 7 and 30×18mm in S6 (Fig. 1a, b). We checked some tumor markers including alpha-fetoprotein, des-gamma-carboxy prothrombin, carcinoembryonic antigen, carbohydrate antigen 19-9, and prostate specific antigen, and they were all within normal ranges (Table 1).

**Table 1:** Laboratory data on the first tumor biopsy

WBC	6,100	/μl	Na	131	mEq/l
RBC	44,900	/μl	K	4.8	mEq/l
Hb	13.9	g/dl	Cl	93	mEq/l
Ht	40.1	%	Ca	9.4	mg/dl
Plt	238,000	/μl	BUN	16.3	mg/dl
PT-%	>100	%	Cr	0.84	mg/dl
TP	7.7	g/dl	CRP	<0.1	mg/dl
Alb	4.5	g/dl			
T.Bil	0.8	mg/dl	AFP	3.7	ng/ml
AST	18	IU/l	DCP	18	mAU/ml
ALT	19	IU/l	CEA	1.9	ng/ml
LDH	264	IU/l	CA 19-9	6.9	U/ml
ALP	127	IU/l	PSA	0.26	ng/ml
Gamma-GTP	68	IU/l			
Glu	75	mg/dl	HBsAg	(–)	
BUN	16.3	mg/dl	HCV-Ab	(–)	
Cr	0.84	mg/dl			

*AFP* alpha-fetoprotein, *Alb* albumin, *ALP* alkaline phosphatase, *ALT* alanine aminotransferase, *AST* aspartate aminotransferase, *BUN* blood urea nitrogen, *Ca* calcium, *CA 19-9* carbohydrate antigen 19-9, *CEA* carcinoembryonic antigen, *Cl* chloride, *Cr* creatinine, *CRP* C-reactive protein, *DCP* des-gamma-carboxy prothrombin, *gamma-GTP* gamma-glutamyl transpeptidase, *Glu* glucose, *Hb* hemoglobin, *HBsAg* hepatitis B surface antigen; *HCV-Ab* hepatitis C virus antibodies; *Ht* hematocrit; *K* potassium; *LDH* lactate dehydrogenase; *Na* sodium; *Plt* platelet; *PSA* prostate specific antigen; *PT-%* prothrombin time percent; *RBC* red blood cell; *T.Bil* total bilirubin; *TP* total protein; *WBC* white blood cell

**Fig. 2:**
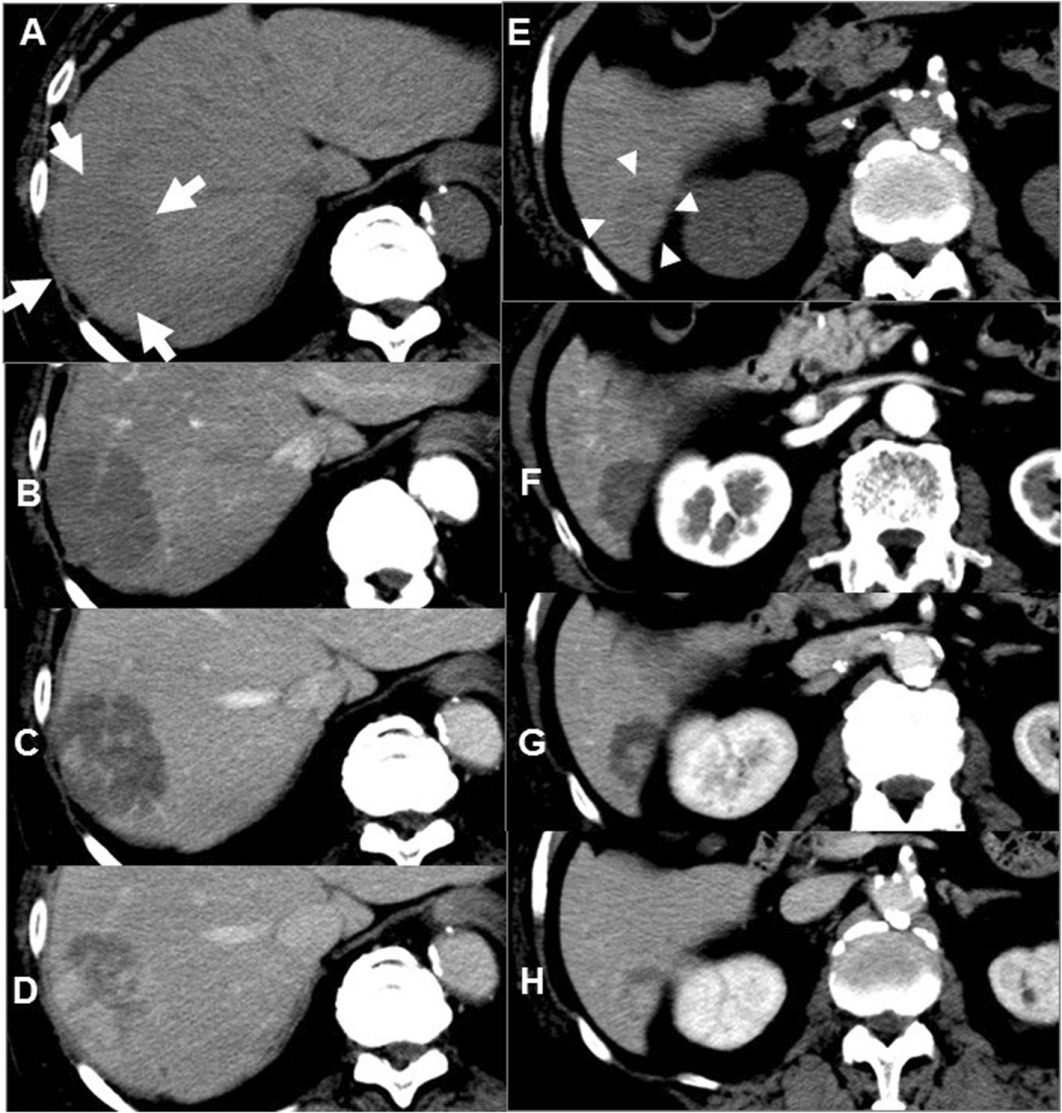
Abdominal computed tomography imaging (**a–d**: segment 7, **e–h**: segment 6). **a** (white arrows), **e** (white arrowheads) Plain phases. **b, f** Arterial phases. **c, g** Portal phases. **d, h** Delayed phases. **a, e** Both tumors are isodense before contrast injections, (**b, f**) and show hypoenhancement in the arterial phase. **c, g** In the portal venous phase, the tumor in segment 7 shows heterogeneous enhancement, and the tumor in segment 6 shows central enhancement. **d, h** In the delayed phase, the tumor in segment 7 shows more heterogeneous enhancement, and the tumor in segment 6 shows more central enhancement

**Fig. 3:**
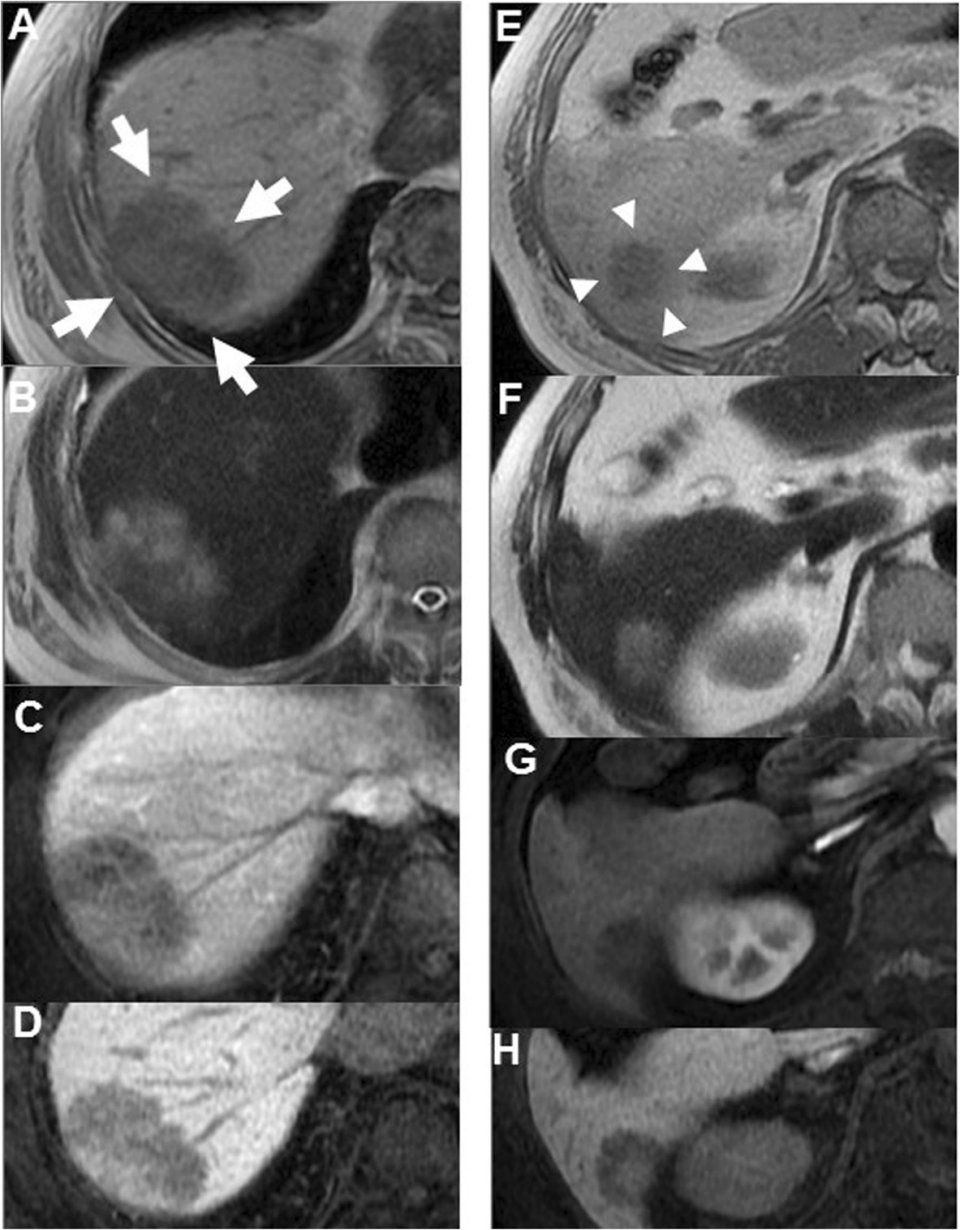
Abdominal contrast-enhanced magnetic resonance imaging (**a–d**: segment 7, **e–h**: segment 6). **a** (white arrows), **e** (white arrowheads) T1-weighted image. **b, f** T2-weighted image. **c, g** Arterial phases on T1-weighted image. **d, h** Parenchymal phases on T1-weighted image. **a, b, e,** and **f** Both liver tumors show the same intensity patterns on T1-weighted image and T2-weighted image. **c, g** However, in the arterial phase on T1-weighted image, the tumor in segment 7 shows heterointensity, but the tumor in segment 6 shows central enhancement. **d, h** In the parenchymal phase of T1-weighted image, the tumor in segment 7 shows more heterointensity, and the tumor in segment 6 shows more central enhancement

Hepatic dynamic computed tomography (CT) indicated the presence of liver tumors (Fig. 2a–h). Both tumors were isodense before contrast injection (Fig. 2a, e) and showed hypoenhancement in the arterial phase (Fig. 2b, f). Afterward in the portal venous phase, the tumor in S7 showed heterogeneous enhancement, and the tumor in S6 showed central enhancement (Fig. 2c, g). Furthermore, in the delayed phase, the tumor in S7 showed more heterogeneous enhancement, and the tumor in S6 showed more central enhancement (Fig. 2d, h). The abdominal magnetic resonance image (MRI) showed hypointense tumors on the T1-weighted image (WI) and hyperintense tumors on T2WI in both tumors (Fig. 3a, b, e, and f). In the arterial phase of T1WI, the tumor in S7 showed heterointensity, but the tumor in S6 showed central enhancement (Fig. 3c, g). Like the CT image, in the parenchymal phase of T1WI, the tumor in S7 showed more heterointensity, and the tumor in S6 showed more central enhancement (Fig. 3d, h). The differential diagnoses after CT and MRI imaging examinations included metastatic liver tumor, inflammatory pseudotumor, epithelioid hemangioendothelioma, and hepatic angiosarcoma.

**Fig. 4:**
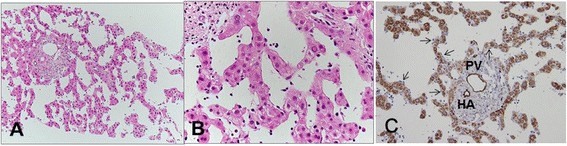
The first needle biopsy. **a** The specimen shows marked sinusoidal dilatation with cystic cavity formation (hematoxylin and eosin staining, ×100). **b** Endothelial cells lining these spaces are flat, and no cellular atypia is identified. Liver cell plates are rather atrophic (hematoxylin and eosin staining, ×400). **c** Immunostaining shows the endothelium of the portal vein (*PV*) and the hepatic artery (*HA*) as positive for CD34, but the sinusoidal endothelium (*arrows*) is negative (×200). Intracytoplasmic granular staining of hepatocytes is nonspecific

In June 2012, we carefully performed the first liver biopsy because he had anticoagulation therapy for an old myocardial infarction. Histological results showed marked sinusoidal dilatation throughout the lobule with cystic cavity formation. The endothelial cells lining these spaces were flat, and no cellular atypia was identified. Liver cell plates were rather atrophic. There was no evidence of malignancy (Fig. 4a, b).

CD34 immunostaining was negative in the sinusoidal endothelium (Fig. 4c). We were able to exclude the differential diagnosis based on typical histological results including CD34 immunostaining, which is a useful marker to distinguish vascular tumors, such as hemangiomas, from PH [7]. We decided to treat him conservatively with regular follow-ups.

**Fig. 5:**
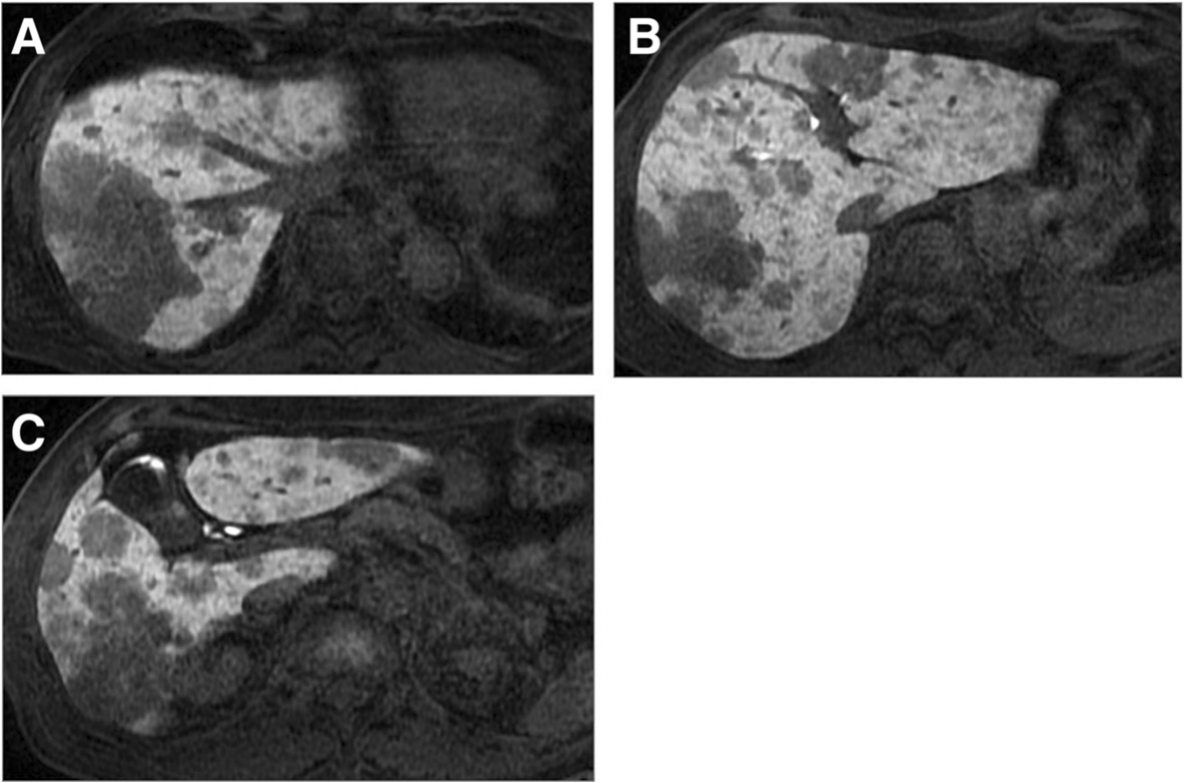
Abdominal contrast-enhanced magnetic resonance imaging before the second tumor biopsy. **a–c** Some tumors show heterointensity, and other tumors show central enhancement in the parenchymal phase of T1-weighted image

**Fig. 6:**
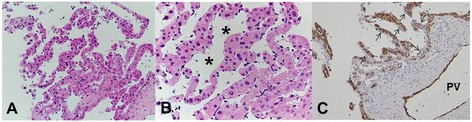
The second needle biopsy. **a** The specimen shows almost the same findings as the previous biopsy (hematoxylin and eosin staining, ×100). **b** Although slight anisonucleosis and chromatin increase of dilated sinusoids-lining endothelium* was observed, there was no convincing evidence of angiosarcoma (hematoxylin and eosin staining, ×400). **c** Sinusoidal endothelium (*arrows*) was negative for CD34 immunostaining. *PV* portal vein

Afterward, these tumors grew rapidly, and the number of tumors increased and disseminated throughout his whole liver (Fig. 5a–c). He remained asymptomatic. In July 2013, we performed a second tumor biopsy. The histological findings were almost the same as those in the previous biopsy. Although slight anisonucleosis and chromatin increase of dilated sinusoids-lining endothelium* were seen, there was no convincing evidence of angiosarcoma (Fig. 6a, b). No CD34 expression was revealed in the sinusoidal endothelium or in the previous biopsy (Fig. 6c).

The patient was reconfirmed as having PH of the liver. We treated him conservatively because he was relatively advanced in age in addition to having several confounding diseases.

## Discussion

To the best of our knowledge, this is the first report of a case in which PH disseminated rapidly to the whole liver in a patient with prostate cancer who had been treated with an LH-RH agonist. PH is a benign tumor-like lesion; however, it is difficult to diagnose accurately without a tumor biopsy because its clinical and radiological characteristics are nonspecific [8].

Regarding the history of the PH diagnoses, in 1861, Wagner first used the term, “pelios,” which in Greek means blackish-bluish with contusion, to describe the gross appearance of the lesions on the cut surface of the liver [2]. Before the 1970s, PH was mainly diagnosed by autopsy. It was later seen in patients with tuberculosis, disseminated malignancies, and hematological disorders [3]. Furthermore, recently PH was considered to be related to HIV [4], immunosuppressive drugs after kidney transplantation, and the administration of oral contraceptives [5]. In the present case, our patient had no immunodeficiency diseases and, therefore, was not given any immunosuppressive drugs. Furthermore, LH-RH agonist injections have been shown to have an opposite effect to oral contraceptives, which contain estrogen and progestin; therefore there might be a causal relationship between the two. However, there might be a relationship between PH and prostate cancer as was previously reported [9]. In the present case, the patient was asymptomatic and had normal laboratory data. It is, therefore, necessary to consider the typical symptoms and laboratory data for PH.

In general, the symptoms and the laboratory data in PH depend on the disease processes. There is no typical tumor marker of PH [8]. However, it is currently considered that PH is related to HIV infection [4], agents administered after kidney transplantation [5, 10], and the administration of oral contraceptives [6]. Furthermore, PH is occasionally found in patients who have worsened liver function [8]. Therefore, when faced with multiple liver tumors accompanied by atypical radiological images, PH should be considered.

In imaging studies, various dynamic enhanced patterns are characteristic. On dynamic contrast-enhanced abdominal CT images, PH shows an isodense pattern before contrast injection; however, PH shows various enhancement patterns after contrast injection [11–13]. Indeed, both of the tumors in this case showed hypoenhancement in the arterial phase. However, in the portal venous phase, one showed heterogeneous enhancement, and the other showed central enhancement. Furthermore, in the delayed phase, one showed more heterogeneous enhancement, and the other showed more central enhancement. Abdominal MRI showed hypointense tumors on T1WI and hyperintense tumors on T2WI in both tumors. However, as in CT images, enhanced MRI shows various enhanced patterns after contrast injection. Therefore, it is exceedingly difficult to accurately diagnose PH with only imaging studies [12, 13].

Histological information is necessary to accurately diagnose PH. A parenchymal type and a phlebectatic type have been reported among the histologic types [8]. The parenchymal type is characterized by the congestion of irregular cavities that are neither lined by sinusoidal cells nor fibrous tissue with the adjacent hepatic tissue occasionally displaying liver cell necrosis [8]. The phlebectatic type is characterized by cavities lined by endothelium. The present case is of the phlebectatic type. CD34 is a useful marker of hematopoietic progenitor cells and endothelial cells to distinguish hemangioma from PH (CD34 positive and negative, respectively) [7]. As expected, the endothelium of this present case was negative for CD34. Furthermore, the bacillary form of PH is caused by infection, mostly occurring in immunocompromised cases. It contains clumps of organisms, such as *Bartonella henselae* or *Bartonella quintana*, which can be identified using the Warthin–Starry stain [7]. No organisms were detected in the liver tissue in this case. Various important findings can be obtained by liver biopsy. However, because they could cause abdominal bleeding, needle biopsies for PH should be performed only in cases in which malignant disease cannot be ruled out by other means [1].

There is no specific treatment for PH because it is not a malignant tumor. Asymptomatic patients and those who have progressive tumors may be conservatively treated with regular follow-ups. Some lesions may disappear spontaneously after withdrawal of possibly related chemicals and medications [7, 14]. In addition, in patients with liver failure complications, liver transplantation may be considered. But, especially with PH, as in every case, the therapeutic options should always be decided depending upon the patient’s condition and situation.

## Conclusions

PH is a rare hepatic benign tumor. However, PH should be considered in cases presenting with multiple unknown liver tumors that are revealed as atypical on radiological images.

## Consent

Written informed consent was obtained from the patient for publication of this case report and any accompanying images. A copy of the written consent is available for review by the Editor-in-Chief of this journal.
